# An ecological study on the relationship between supply of beds in long-term care institutions in Italy and potential care needs for the elderly

**DOI:** 10.1186/1472-6963-9-174

**Published:** 2009-09-24

**Authors:** Gianfranco Damiani, Simona C Colosimo, Lorella Sicuro, Alessandra Burgio, Alessandra Battisti, Alessandro Solipaca, Giordana Baldassarre, Roberta Crialesi, Giulia Milan, Tiziana Tamburrano, Walter Ricciardi

**Affiliations:** 1 Department of Public Health, Università Cattolica Sacro Cuore, Rome, Italy; 2 Health and Assistance Unit, Italian National Institute of Statistics, Istat, Rome, Italy

## Abstract

**Background:**

The ageing population in Europe is putting an ever increasing demand on the long-term care (LTC) services provided by these countries. This study analyses the relationship between the LTC institutional supply of beds and potential care needs, taking into account the social and health context, the supply of complementary and alternative services, along with informal care.

**Methods:**

An observational, cross-sectional, ecological study was carried out. Statistical data were obtained from the Italian National Institute of Statistics and Ministry of Health. Indicators, regarding 5 areas (Supply of beds in long term care institutions, Potential care needs, Social and health context, Complementary and alternative services for the elderly, Informal care), were calculated at Local Health Unit (LHU) level and referred to 2004.

Two indicators were specifically used to measure supply of beds in long term care institutions and potential care needs for the elderly. Their values were grouped in tertiles. LHU were classified according to the combination of tertiles in three groups: A. High level of supply of beds in long term care institutions associated with low level of potential care needs; B. Low level of supply of beds in long term care institutions associated with high level of potential care needs; C. Balanced level of supply of beds in long term care institutions with potential care needs. For each group the indicators of 5 areas were analysed.

The Index Number (IN) was calculated for each of these indicators.

**Results:**

Specific factors that need to be carefully considered were highlighted in each of the three defined groups. The highest level of alternative services such as long-stay hospital discharges in residence region (IN = 125), home care recipients (HCR) (IN = 123.8) were reported for Group A. This group included North regions. The highest level of inappropriate hospital discharges in (IN = 124.1) and out (IN = 155.8) the residence region, the highest value of families who received help (IN = 106.4) and the lowest level of HCR (IN = 68.7) were found in Group B. South regions belong to this group. The highest level of families paying a caregiver (IN = 115.8) was shown in Group C. Central regions are included in third group.

**Conclusion:**

Supply of beds in long term care institutions substantially differs across Italian regions, showing in every scenario some imbalances between potential care needs and other studied factors. Our study suggests the need of a comprehensive rethinking of care delivery "system".

## Background

The developed world's population is aging because of the trends of increasing in life expectancy and decreasing of fertility rates. These demographic changes result in an increasing share of old and very old people, leading to new patterns of morbidity and mortality, such as the increasing number of degenerative and often multiple and chronic diseases. These trends are predicting the increase of demand on long-term care (LTC) services [1].

The Organization for Economic Cooperation and Development (OECD) has defined long-term care as a policy issue that brings together a range of services for persons who are dependent on help with basic Activities of Daily Living (ADL) over an extended period of time. It also includes Instrumental activities of daily living (IADL) that are activities related to independent living and include preparing meals, managing money, shopping for groceries or personal items, performing light or heavy housework, and using a telephone [2]. LTC is usually provided to persons with physical or mental disability, the frail elderly and particular groups that need support in conducting their daily life activities and seem unable to care for themselves without the help of another person [1]. LTC includes a variety of medical and non-medical services addressed to people with chronic health conditions and/or physical disabilities.

LTC involves formal care (the amount of care supplied by the health and social care systems - regarding these systems in Italy see Additional file 1) and informal care (the amount of care and assistance supplied by close relatives, neighbours and caregivers paid out of pocket) [3]. According to Saltman LTC services can be classified in three groups: home care, including clinical and social activities provided by formal or informal providers; sheltered housing or old-age-homes operated or paid for by public municipalities, non-profit voluntary, or for-profit organizations and nursing homes providing intensive care [4].

While the overall need for LTC services is expected to grow substantially, these types of services and resource requirements in financing their delivery can differ among developed countries. Thus in Northern Europe the older people receiving institutional care make up 12% of all elders, while in Southern Europe this number is only 3% for Italy and less than 1% for Greece [5].

Moreover, in providing long-term care for older people, lots of developed countries, especially in Western Europe and in Scandinavia, have been moving from institutional care to community or home care provision encouraging informal family support, implementing direct payments and integrating housing, health and social care services [6,7].

In order to explain the different strategies adopted in the financing and delivery of long-term care services several factors have to be considered, such as: different welfare regimes, different family ethics and old age policies, but also general social trends, the reduced informal support for families by increased economic migration and women's increased labour market participation [5].

Hence, it is a priority from the policy making point of view to study the actual role of residential LTC and specifically to assess the relationship between supply of beds in long term care institutions and potential care needs for the elderly. With regards to this relationship, Ribbe compared the long-term care systems in 10 countries showing that there is no relationship between the ageing status of a country and the number of nursing home beds [8]. In recent years, Huynh also examined the association between the increasing number of older persons and disabled people and the number of nursing homes beds available in each region of Kentucky. This study showed a significant mismatch between the number of available long-term beds and the potential needs [9].

In Italy, as in other countries, the rapid population ageing is coupled with the recent challenging evolution of Italian National Health Service (NHS) which prompts the policy-makers to require more information on long-term services so as to adopt specific strategies to meet increasing health needs and contain future expenditures connected with aging. The purpose of this study is to perform an analysis of the relationship between the long-term care institutional supply of beds in Italy and potential care needs, taking into account the social and health context, the supply of complementary and alternative services, along with informal care.

## Methods

### Study design and Data sources

An observational, cross-sectional and ecological study was conducted on Italian elderly (65 years and over population) referred to 2004.

Official statistical data provided by the National Institute of Statistics and the Ministry of Health were analyzed to calculate indicators.

Data were collected from the following sources:

*Health conditions and recourse to health services *[A].

*Households and social subjects *[B].

*Residential Care Institutions *[C].

*Census Survey on intervention and social services provided by single and associated municipalities*. [D].

*Resident population by age, gender and marital status *[E].

*Hospital discharge register *[F].

*Home care for the elderly *[G].

An analytical description of each afore mentioned source is detailed in Additional file 2.

### Selection Criteria

**Long Term Care Institutions for the elderly **were selected concerning health and social patterns. Regarding skilled nursing facilities and nursing homes, all institutions were included. From adult foster care facilities, social rehabilitation facilities, assisted living facilities, residential care facilities for the elderly, only those institutions having health personnel employed (physicians, psychologists, medical attendants, physiotherapists, speech therapists and other personnel for rehabilitation) and those who had NHS's funding were included. Furthermore, in the case of the first three types of institutions only those with elderly recipients were considered.

As regards **Hospital discharge register**, inappropriate and long stay discharges were selected. In particular the inappropriateness of hospital care was defined as an admission which results and benefits could have been obtained at a lower level of care [10].

Complying with this view, we referred to a list of 43 codes of medical and surgical Diagnosis Related Groups (DRG) for inpatients, enacted by an Italian governmental decree (DPCM 29/11/2001). These 43 DRG could be treated, on the basis of a cost saving approach, in other settings different from hospital inpatient care.

Regarding long-stay discharges, we referred to inpatients admitted to acute care hospitals with a length of stay ≥ 30 days.

### Indicators

Indicators were grouped into 5 different areas:

(Letter in brackets "[]" refers to the data source described above)

#### Supply of beds in long term care institutions for the elderly

The rate of *Beds in long term care institutions for the elderly *was calculated per 10,000 population aged 65 and over. [C]

#### Potential care needs for the elderly

*Prevalence of disability*: elderly people with at least one disability - difficulties in seeing, hearing, movement, function of activities of daily living to total resident elderly population- [11,12]. [A]

#### Social and health context

Following rates were calculated per 10,000 population aged 65 and over:

*Perceived bad health status*: elderly declaring feeling bad or very bad. [A]

*Educational level*: elderly without formal education or with primary or secondary school. [A]

*Elderly living alone*: elderly living alone. [A]

*Scarce economic resources of family*: elderly considering inadequate their economic resources. [A]

*Unsatisfied home care need*: elderly with unsatisfied demand of home care. [A]

Another rate *Resident population aged 80 and over *(resident population aged 80 and over to the overall population [E]) completed this set.

#### Complementary and alternative services for the elderly

All rates were calculated per 10,000 population aged 65 and over:

*Elderly receiving social and health payments*: elderly who receive social and health payments (voucher or care benefits). [D]

*Users of adult day-care centres*: elderly users of adult day-care centres. [D]

*Net expenditure for social and health services*: net expenditure (Euros) per capita for three elderly services (social visiting services, social and health payments, adult day-care centres). [D]

*Inappropriate hospitalisation in residence region*: inappropriate hospital discharges of elderly in residence region. [F]

*Inappropriate hospitalisation in region different from residence*: inappropriate hospital discharges of elderly in region different from their residence. [F]

*Long-stay hospitalisation in residence region*: long-stay hospital discharges of elderly in residence region. [F]

*Long-stay hospitalisation in region different from residence*: long-stay hospital discharges of elderly in region different from their residence. [F]

*Home care recipients*: elderly users of health home-care. [G]

#### Informal care

All rates were calculated per 10,000 families with at least one elderly:

*Families with a social network*: families with at least one elderly supported by a social network composed by relatives, friends and neighbours. [B]

*Families who received help*: families with at least one elderly who received economic, social and/or health aids in the last four weeks from friends or relatives not living in the same apartment. [B]

*Families paying a caregiver*: families with at least one elderly paying a caregiver. [B]

### Statistical analysis

In the first part of the study, descriptive analyses were conducted to evaluate the regional distributions of the main indicators.

In the second part of the analysis indicators were calculated at minimum geographical level used in the survey. Indeed, they had different territorial level, both for conceptual requirement because of the health and social care organizational model and for data availability.

All selected indicators were assigned to their specific LHU, for example if an indicator was available only at regional level its value was assigned to all LHU belonging to the considered region.

The LHU territorial dimension was detected because it represents a key point of connection between long term care institutions and population needs. These two dimensions were analyzed jointly to classify the LHU.

For the first dimension selected indicator was the supply of beds in long term care institutions for the elderly, while for the second dimension the prevalence of disability was chosen as a proxy of potential care needs for the elderly because this group represents an appropriate target for the long term care institutions.

Distributions of these two indicators were divided in three parts, using the tertiles of the distribution and moving, from the first to the third, towards rising level of each measured dimension - low (L), middle (M), high (H) [13].

Then LHU were classified according to the combination of the two indicators into three groups.

Each group represented the comparison, based on ordinal scale, between supply of beds in long term care institutions and potential care needs for the elderly. In this way the level of supply was considered balanced with the level of potential care needs if LHU assumed the same value of the ordinal scale (LL, MM, HH combinations). On the other side the comparison was considered inconsistent, by defect or by excess, if LHU assumed different values of the ordinal scale (HM, HL, ML, LM, LH, MH combinations).

The three groups were composed as follow (Table 1):

**Table 1:** Combination between levels of supply of beds in long term care institutions and potential care needs for the elderly.

		**Prevalence of disability**		
		**L** **(Low-I tertile)**	**M** **(Middle-II tertile)**	**H** **(High-III Tertile)**

**Rate of beds in** **long term care** **institutions for** **the elderly**	**L** (Low-I tertile)	LL	LM	LH
	
	**M** (Middle-II tertile)	ML	MM	MH
	
	**H** (High-III tertile)	HL	HM	HH

- Group A: LHU with high level of supply of beds in long term care institutions coupled with low level of potential care needs for the elderly (HM, HL, ML);

- Group B: LHU with low level of supply of beds in long term care institutions coupled with high level of potential care needs for the elderly (LM, LH, MH);

- Group C: LHU with balanced levels of supply of beds in long term care institutions and potential care needs for the elderly (LL, MM, HH).

The Index Number (IN) was calculated for each indicator of social and health context, complementary and alternative services of long-term care and informal care, to analyze the scenario for each of the three groups just mentioned.

The IN is the ratio between the mean value of each indicator in a single LHU group and the mean value of the same indicator in all the LHU, multiplied by 100. Then the IN for Italy is equal to 100. One-way Analysis of Variance (ANOVA) test was used to evaluate if there were statistically significant differences (with a p-value < 0.05) among the three selected groups.

## Results

In 2004 Italian elderly were 11,254,000, with an increase of 10.1% compared to 1999, and represented 20% of the national population.

### Supply of beds in long term care institutions for the elderly

**Figure 1:**
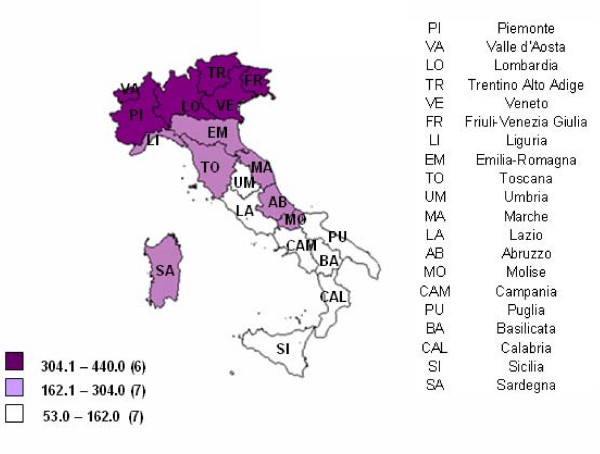
**Beds in long term care institutions for the elderly by Region. 2004 year. (Rates per 10,000 elderly)**. Graduation of colours refers to tertiles of Regions. In brackets the number of regions belonging to each tertile. Data Source: Residential care institutions survey.

Long term care institutions for elderly were 3,952 with 251,566 available beds. A wide regional variability was observed. In the North it ranged from 263 beds per 10,000 elderly in Emilia Romagna to 530 beds in Trento. In the South values differed from 54 to 179 beds per 10,000 elderly in Campania and Molise respectively (Figure 1).

### Potential care needs for the elderly

**Figure 2:**
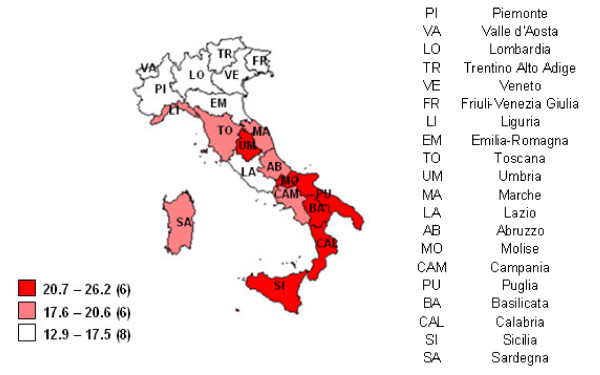
**Percentage of elderly people with at least one disability by Region. 2004 year**. Graduation of colours refers to tertiles of Regions. In brackets of legend there are the number of regions in each tertile. Disability is defined as difficulties in seeing, hearing, movement, functions of activities of daily living. Data Source: Health conditions and recourse to health services survey.

The epidemiological profile among elderly was mainly characterized by disability (prevalence = 18.7%). This prevalence of disability showed a geographical gradient from the North to the South of Italy: the minimum was in Bolzano (North) (12%) and the maximum was in Sicilia (South) (26%) (Figure 2).

### Social and Health Context

Among elderly, 20% declared Perceived bad health status (16% of females and 24% of males). The value of this indicator ranged from 10% in Trento (North) to 28% in Calabria (South).

Regarding Educational level 82% of males and 89% of females aged 65 and over had primary education or lower secondary education. With respect to an average of 86% at national level the highest value 93% was observed in Basilicata (South).

Women lived more frequently alone than men, respectively Elderly living alone was equal to 37% for women, vs. 14% for men. This condition was mainly (36%) reported in Valle d'Aosta (North). On the contrary the lowest percentage (21%) was in Marche (Centre).

Elderly considering Scarce economic resources of family were 37%, ranging from 19% to 45% in Valle d'Aosta (North) and Sicilia (South) respectively.

Unsatisfied home care need affected 14% of elderly (17% females and 11% males) showing a geographical gradient with the highest peak (24%) in Calabria (South) and the lowest one (6%) in Bolzano (North).

Resident population aged 80 and over were 2,832,015 representing the 4.9% of the total population (6% females and 3% males). The extent of geographical variability was pointed out from 3% in Campania (South) to 7% in Liguria (North).

### Complementary and alternative services for the elderly

Elderly receiving social and health payments were 73 per 10,000. At regional level a wide variability was observed: none received social and health payments in Valle d'Aosta (North), while 455 elderly per 10,000 received social and health payments in Bolzano (North).

A very high regional variability was found for users of day care centres too: they were 100 per 10,000 at national level with the lowest rate 5 in Basilicata (South) and the highest peak 424 in Abruzzo (South).

A North-South gradient was observed for the Net expenditure for social and health services. Bolzano (North) and Valle d'Aosta (North) had the highest expenditure with respectively 375 and 354 euros per capita and Calabria (South) the lowest value with 7.60 euros (the national average was 48.50 euros).

Inappropriate hospital discharges among elderly were about 523 thousands, of which 5% occurred in a region different from residence of the patient. This percentage increased in small regions (Valle d'Aosta (North) 28%, Basilicata (South) 20%, Trento (North) 13%, Molise (South) 12%). The rate was equal to 465 hospital discharges per 10,000 elderly, with the lowest value of 221 in Valle d'Aosta (North) and the highest value of 911 in Sardegna (South).

Long-stay hospital discharges were about 122 thousands, of which 7% occurred in a region different from residence of the patient. There was a wide regional variation of this percentage from 36% in Basilicata (South) to 2% in Lazio (Centre) and Veneto (North). The rate was equal to 109 hospital discharges per 10,000 elderly with the lowest value of 39 in Sicilia (South) and the highest value of 221 in Lazio (Centre).

Regarding elderly people treated at home, there were about 278 Home care recipients per 10,000 elderly (313 thousands). Friuli Venezia Giulia (North) (770) and Molise (South) (674) presented the highest values, while Valle d'Aosta (North) (20) and Bolzano (North) (25) had the lowest rates.

### Informal care

Families with a social network were 17%. Social network seemed to be more developed in Molise (South) and in Toscana (Centre) (22%) while region with the lowest percentage was Abruzzo (South) (11%).

Families who received help by friends or relatives were 18%. The peak value was in Sardegna (South) (22%) and the lowest value was in Valle D'Aosta (North) (13%).

Families paying a caregiver were only 3% with a small variability from 1% in Piemonte (North) to 5% in Abruzzo (South).

### Tertiles analysis

**Figure 3:**
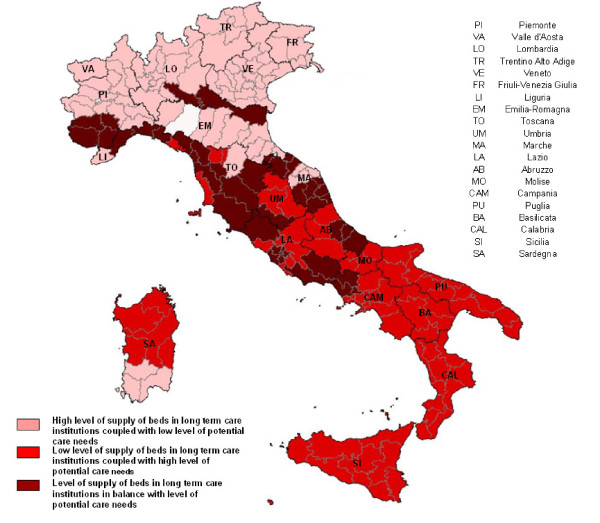
**Partitions of LHU in three groups. 2004 year**. Graduation of colours refers to the three LHU groups. The white LHU, Parma in Emilia Romagna, was not taken into consideration in the analysis because no information on beds in long term care institutions for the elderly was available.

The analysis of the indicators of social and health context, complementary and alternative services of long-term care and informal care, pointed out some relevant differences among the 3 groups coming out from the combination of institutional availability and potential care needs (Table 2). The ANOVA test showed statistically significant differences for each indicator except for Families with a social network (p = 0.146) and Scarce economic resources of family (p = 0.355). Group A showed the highest value of all the alternative services of health and social care such as Elderly receiving social and health payments (IN = 184.6), long-stay hospital discharges in residence region (IN = 125), Home care recipients (IN = 123.8). Although in this group there was the highest number of Elderly living alone (IN = 106.7) there was also the lowest number of elderly with an Unsatisfied home care need (IN = 65.8). Regarding geographical areas, this group included the North Italian regions, in particular all LHU of Bolzano, Veneto and Friuli Venezia Giulia, together with almost all LHU of Emilia Romagna and Lombardia (Table 3 and Figure 3).

**Table 2:** Index numbers by group of LHU. 2004 year.

	**GROUP A**	**GROUP B**	**GROUP C**	**F Fisher**	**P-value**
Perceived bad health status	82.1	119.8	100.4	82.865	< 0.001

Educational level	98.7	101.6	99.8	5.688	0.004

Elderly living alone	106.7	96.4	92.7	11.681	< 0.001

Scarce economic resources of family	97.8	102.4	100.0	1.040	0.355

Unsatisfied home care need	65.8	144.9	88.5	129.407	< 0.001

Resident population aged 80 and over	102.6	89.4	113.6	13.738	< 0.001

Elderly receiving social and health payments	184.6	33.2	43.0	42.515	< 0.001

Users of adult day-care centres	83.7	84.7	163.0	3.787	0.024

Net expenditure for social and health services	153.6	59.4	61.6	16.333	< 0.001

Inappropriate hospitalisation in residence region	86.3	124.1	84.3	18.970	< 0.001

Inappropriate hospitalisation in region different from residence	53.0	155.8	94.6	22.974	< 0.001

Long-stay hospitalisation in residence region	125.0	72.3	97.6	18.678	< 0.001

Long-stay hospitalisation in region different from residence	76.6	125.2	103.2	4.719	0.010

Home care recipients	123.8	68.7	106.7	4.889	0.009

Families with a social network	96.3	102.2	103.1	1.945	0.146

Families who received help	94.6	106.4	98.8	12.256	< 0.001

Families paying a caregiver	80.9	112.4	115.8	23.424	< 0.001

**Table 3:** Partition of the LHU: description of variables and composition of each group. 2004 year.

	**GROUP A** **79 LHU**	**GROUP B** **71 LHU**	**GROUP C** **38 LHU**
REGION (a)	Piemonte (15/19)	Liguria (1/5)	Piemonte (4/19)
	Valle d'Aosta (1/1)	Toscana (3/12)	Lombardia (2/15)
	Lombardia (13/15)	Umbria (3/4)	Liguria (3/5)
	Bolzano (4/4)	Lazio (4/9)	Emilia Romagna (1/10)
	Trento (1/1)	Abruzzo (3/6)	Toscana (8/12)
	Veneto (21/21)	Molise (4/4)	Umbria (1/4)
	Friuli-Venezia Giulia (6/6)	Campania (11/13)	Marche (9/13)
	Liguria (1/5)	Puglia (12/12)	Lazio (5/9)
	Emilia Romagna (9/10)	Basilicata (5/5)	Abruzzo (3/6)
	Toscana (1/12)	Calabria (11/11)	Campania (2/13)
	Marche (4/13)	Sicilia (9/9)	
	Sardegna (3/8)	Sardegna (5/8)	

(a) in brackets number of LHU of each group/total number of LHU in the region

Group B showed the highest level of Inappropriate hospital discharges in (IN = 124.1) and out (IN = 155.8) the residence region and also the highest value of Families who received help (IN = 106.4). The group was characterized also by the highest value of Unsatisfied home care needs (IN = 144.9), the lowest value of Home care recipients (IN = 68.7) and the lowest Net expenditure for social and health services (IN = 59.4). LHU in this group belonged mainly to the South of Italy (Table 3 and Figure 3).

Group C was characterized by the highest level of Users of adult day-care centres (IN = 163.0) and of Families paying a caregiver (IN = 115.8); on the contrary, Inappropriate hospital discharges in residence region had the lowest value (IN = 84.3). Most LHU of Liguria, Toscana and Marche belonged to the third group (Table 3 and Figure 3).

## Discussion and Conclusion

This study provides policy makers a feasible and objective methodological approach that makes possible to take measurements using set of indicators, which could be easily retrieved from administrative and statistical data. It focuses on the relationship between supply of beds in long term care institutions and potential care needs for the elderly, but, taking into account also the main features of the social and health context, the supply of complementary and alternative services, including both home care and informal care, an evaluation-oriented contribution can be provided to support the decision making process for the long term care of the elderly.

The application of this approach to Italian context showed how supply of beds in long term care institutions for the elderly substantially varies across Italian regions. The analysis revealed a certain degree of incongruity in the groups of selected LHU. The level of potential care needs follows a North-South geographical gradient that might be partially explained by a similar geographical distribution of level of education (percentage of population with primary school certificate: 27.2% in the North and 31.2% in the South) [14] and income (family average net income: 36,642 euros in the North and 26,627 euros in the South) [15-18].

These socioeconomic conditions are also strongly related to individual behaviors (physical activity, nutritional habits, smoking, drinking) [19] that in Italy are distributed on the basis of geographical gradient in many cases [16,20]. These factors might play an important role in determining health care needs [21-23].

Furthermore, cultural differences in self-definition of ill-health might also affect this geographical distribution of health needs.

In group A, a low level of potential care needs seems to be mismatched with the high level of supply of beds in long term care institutions coupled with home care recipients and other alternative services.

This may be explained by the large numbers of elderly living alone and the high employment rates in Northern households, included women. [24]. These factors facilitate the recourse to the institutionalization. Such a situation was promptly intercepted by private LTC provider investors determining the development of bricks-and-mortar housing services. Private entrepreneurism was also active in delivering formal home care services in the Northern Regions. Regarding very low availability of residential services in Southern Italy, as showed in Group B, it might reflect some cultural and religious factors. In fact, in Southern Italy the care for elderly is viewed as natural responsibility of the family.

For these reasons the families in Southern Italy might be reluctant to institutionalize their disabled relatives and want to keep them at home as long as possible. This sense of responsibility for the care of elderly that traditionally characterizes Southern Italy families might also explain the low level of home care recipients in Group B. Therefore, these potential care needs meet a response trough the informal care provided by relatives or caregivers supporting the family.

Another factor that might explain the lack of elderly services is the use of acute care hospital beds [25,24]. In those years the hospitalization rate was 140.7 admissions per 1,000 residents in the South with a marked difference compared with 128.4 in the North. This can explain inappropriate hospital discharges, clearly evident in Group B.

In Group C, despite the balance between supply of beds in long term care institutions and potential care needs for the elderly, there was a high level of family paying a care giver. This might be partly affected by the highest share of very old who could need more care, but further studies need to be conducted to understand thoroughly the reasons of this phenomenon.

Since 1993 Burke has suggested to policymakers to take into account the decreasing numbers of elderly people with disabilities by redirecting long term care resources toward quality community- based care [26]. Currently in Italy the age-standardized rate of disabled elderly had been declined between 1994 and 2004-2005, from 21.7% to 18.8% [27].

In addition, already in the 1980s, facing with a rapidly increasing elderly population and soaring costs of health and long-term care services, many governments as Sweden, Denmark, the Netherlands and Great Britain discouraged the building of additional nursing homes and turned to new models of home and community-based care testing different approaches for providing high quality, low-cost care in the home and in the community [28].

Recent evidence suggests that disabled and older people tend not to want institutional care, and families and other informal carers strongly prefer to continue to care for their dependent family members in a friendly environment such as their own homes and local communities [29]. Thus in South Korea, due to specific characteristics of healthcare system and to cultural attitudes toward end-of-life care, home care agencies rather than nursing home provide care to severely impaired patients [30,31].

Therefore, further issues might be related to study the impact of other individual factors on the supply system of LTC including not only the elderly patients' health condition, but also the family caregiver's needs and preferences [32].

This evidence coupled with the results of our study suggests to redirect more properly the "system" of care delivery. In particular, these findings might help policymakers to understand the causes of the greater need that is unmet, and the degree to which they might develop programs to reduce that need rather than just build up more expensive services, especially long term care institutions.

On this basis, a priority seems to be not only to focus on the specific modalities of LTC provision, but to build an adequate form of managed and integrated care for the entire system [33]. An adequate local improvement of "community care access center" might be a proper answer to this issue [34,35]. In fact, the "community care access center" could better define: the needs of elderly trough a multidimensional evaluation; the managed care plan production to create integration among social and health services and informal care to better guarantee the continuity of care; the role of a specific case manager who might guarantee more proper and coordinated levels of care; the development of multi-professional teams among health professionals, health and social caregivers and informal carers.

These efforts to improve LTC should be adequately supported by dedicated funding as suggested by European Union. In fact, Member States are committed to ensure accessible, high-quality and sustainable health care and long-term care by promoting a rational use of resources notably through appropriate incentives for users and providers, good governance and coordination between care systems and public and/or private institutions [2].

## Abbreviations

LTC: Long-Term care; LHU: Local Health Unit; IN: Index Number; ADI: home care; OECD: Organization for Economic Cooperation and Development; ADL: Activity of Daily Living; IADL: Instrumental Activity of Daily Living; NHS: National Health Service; DRG: Diagnosis-Related Group.

## Competing interests

The authors declare that they have no competing interests.

## Authors' contributions

GD, AS, RC, ABU and WR contributed to the conception of this paper. GD, SCC and ABU conceived the study, participated in its design and drafted the manuscript; AS and ABA conceived the statistical methodology; LS, ABA, GB provided the acquisition of data and performed statistical analyses; GM and TT contributed to the acquisition of data and participated in the study design. All authors have seen and approved the final version. GD and RC had full access to all of the data in the study and take responsibility for the integrity of the data and the accuracy of the data analysis.

## Pre-publication history

The pre-publication history for this paper can be accessed here:



## Supplementary Material

Additional file 1 **Appendix 1**. Italian Health and Social Care System. Click here for file

Additional file 2 **Appendix 2**. Analytical description of sources utilized for calculation of indicators considered. Click here for file
