# Supplementary material for: An ecological study on the relationship between supply of beds in long-term care institutions in Italy and potential care needs for the elderly
Source: BMC Health Serv Res. 2009 Sep 24;9:174. doi: 10.1186/1472-6963-9-174 (PMC2762968; doi:10.1186/1472-6963-9-174)
Supplement: Additional file 1 — Appendix 1. Italian Health and Social Care System. [file 1472-6963-9-174-S1.DOC]

Appendix 1

###### Italian Health and Social Care System

**Health care system** [1, 2]

The Italian National Health Service (NHS) was established in 1978 to guarantee an equal access to essential health care services. These services are provided free of charge or by a co-payment of services. They include general medical and paediatric services, essential drugs and those for chronic diseases, treatments administered during hospitalization, rehabilitation and long-term post acute inpatient care, instrument and laboratory diagnostics, as well as other specialized services for early diagnosis and prevention.

The Italian NHS’s organizational structure consists of three levels: central government; regions and autonomous provinces and local health unit (LHU). The Central Government is responsible for national health planning and annual financial resources. It also has the exclusive power to set the so-called “essential levels of care” (Livelli Essenziali di Assistenza, LEAs), an explicit health benefit package, publicly funded , which must be guaranteed to all residents. Since 2001 several important administrative and organizational responsibilities have transferred from the Central Government to the nineteen Regions and two Autonomous Provinces. The Regions and the Autonomous Provinces organize services that meet the needs of their specific populations, define ways to allocate financial resources to all LHU within their territories, and monitor and assesses their performance. The LHU (nowadays 145 units) form the basic elements of the Italian NHS. Based on the criteria of efficiency and cost effectiveness, the LHU can provide care either directly, through their own facilities, or by paying for the services delivered by providers accredited by Regions, such as independent public and private structures. Patients can freely choose among the public or accredited private providers but can also be treated in the LHU in the area of their residence or in another LHU.

**Social Care System** [3]

The social care sector had a parallel legislative reform. In November 2000, the parliament, after four years of work and discussions, succeeded in passing a general policy law (Law 328/2000) reforming Italian social care system according to universal principles. The reform provides new benefits for people with difficulties (as defined by article 38 of Italian Constitution), such as subsidizing the integrated home care system and the service sector (not-for-profit associations, private structures etc.) Municipalities, Regions and the State are the institutional actors responsible for implementing and furthering the integrated network of social services. Municipalities have managerial functions, and their role is central in the actual delivery of social services. Regions have planning and policy tasks such as: criteria for accrediting, authorizing and supervising public and private social service providers and define the quality requirements for managing and providing services. The Central Government grants financial resources ( National Social Fund) and defines the minimum level of social care (a sort of social benefit package) that every region has to guarantee. Every three years, the Government, together with local authorities, defines the National Social Plan which sets the main objectives of social policy and the activities to be undertaken for non-self-sufficient elderly people, people with disability, children and their families and immigrants. The National Social Plan also implements measures against alcoholism and drug abuse.

**Integration of Health and Social care services**

Since the end of the 20^th^ century the importance of integration between health and social care systems was recognized by the central and local governments. Universalism was defined as the main principle of the Health and the Social care systems. The National Health Planning of 1998-2000 (DPR 23 July 1998) set social and health care integration as a strategic priority not only for the central government but also for the regional government, especially for some areas such as: maternal and child care, disability, drug addiction, elderly, long-term care. The Laws n.328/2000 and n.229/1999 detect the Local Planning (Piano Sociale di Zona- PdZ) and the Program of territorial activity (Programma di attività territoriale-PAT) as instruments to integrate and coordinate social and health care services. They take into account the complementarities of social and health intervention but also the coordination of all private and public organizations operating in this field.

**Main system of financing**

Health and Social care systems are currently financed through a regional tax on productive activities (which had replaced social health insurance contributions in 1997), general taxation collected centrally, various other regional taxes and users’ co-payments, but also through private sources of financing. Italy has two main types of out-of-pocket payments. The first is demand-side cost-sharing: a co-payment for diagnostic procedures, pharmaceuticals and specialist visits. The second is direct payment by users for the purchase of private health care services and over-the-counter drugs.

The progressive move towards fiscal federalism started in 1997, and regional taxes finance most health care expenditure, with general taxation playing a complementary role. In fact, central funding is intended to be used primarily to redistribute resources to the regions with a narrower tax base, to ensure adequate levels of care received by all residents.

**Long Term Care system for the elderly**.

Basically LTC system in Italy is aimed to provide the LEAs related to integrated social and health care at home or in institutional setting. Both are under a public regional funding and governance and this latter action is ruled by the District.

This is a basic organizational unit, belonging to LHU and in charge of primary care targets such as individual prevention activities, patient first contact, chronic care disease management and long term care. The District has the commitment to guarantee each old patient potentially targeted for long term care a multidimensional community-based geriatric evaluation aimed to integrate health and social services. This evaluation is performed by a multidisciplinary team that includes registered nurses, social workers, physiotherapists, patient's general practitioners (GPs) and geriatricians. After this assessment an individualized care plan is worked out and the patient is referred to home or institutional services. At the time of the analysis, these services covered 278 home care recipients per 10,000 elderly and 187 elderly institutional recipients per 10,000. These services for the elderly are provided both by public and private providers. Among these services, integrated home care (Assistenza Domiciliare Integrata-ADI) and skilled nursing facility (Residenza Sanitaria Assistenziale-RSA) are the two major ones.

The ADI should meet all the needs for long-term care, from social interventions (homemaking services, meals on wheels, psychological support, and social worker in home, support in administrative duties) to health care programs (daily nurse monitoring, home health aids, physical and occupational therapy programs).

The RSAs were clearly defined in an Act that was promulgated by the Italian Parliament in 1992 (Progetto Obiettivo Salute dell’ Anziano - Objective Project for the Protection of Elderly Health). This was not a law, but rather a document providing a series of suggestions and recommendations to LHU and it defines RSAs "as the territory facilities aiming at providing health and social services to older persons with functional impairment. The eligibility for admission to the RSA is subordinated to the proven lack of suitable family support that hinders such intervention at home".

Organizational arrangements on the continuity and kind of care for the elderly are decided at regional level especially after the devolution of financial, administrative and organizational responsibilities in 2001.

**References**

1. France G, Taroni F, Donatini A: **The Italian health-care system.** *Health Econ* 2005, **14**: S187-S202.

2. Maio V, Manzoli L: **The Italian Health Care System: W.H.O. ranking versus public perception**. *Pharmacy and Therapeutics* 2002, **27**(6): 301-8.

3. Eurostat - European Observatory on Health Care System - *Health Care System in Transition*. 2001.
