# Supplementary material for: An ecological study on the relationship between supply of beds in long-term care institutions in Italy and potential care needs for the elderly
Source: BMC Health Serv Res. 2009 Sep 24;9:174. doi: 10.1186/1472-6963-9-174 (PMC2762968; doi:10.1186/1472-6963-9-174)
Supplement: Additional file 2 — Appendix 2. Analytical description of sources utilized for calculation of indicators considered. [file 1472-6963-9-174-S2.DOC]

Appendix 2

**Analytical description of sources used in calculation of indicators:**

*Health conditions and recourse to health services* [A]: a five year cross-sectional sample survey on 52,332 households, with 140,011 residents in 1,463 municipalities in Italy. It involves observation of perceived health status, disease symptoms, chronic disability conditions and social determinants of health . This survey is composed of two different questionnaires. The first investigates socio-demographic characteristics, cultural and economic conditions and region of residence. The second is self-compiled and gives information about health related quality of life, lifestyle, access to/use of health care services and non-institutional support systems.

*Households and social subjects* [B]: a five-year cross-sectional sample survey on 19,000 households, with 49,000 residents in Italy. The survey is composed of three different questionnaires. The first and the second investigate different areas of interest such as household facilities and commuters in the household, relationships among relatives; informal and formal aids received on the occurrence of critical events, working careers and social mobility. The third is self-compiled and gives information about life in couples and weddings, leaving the household of origin and life cycle; prolonged stay of young adults within their household.

*Residential Care Institutions* [C]: an annual census of Residential Care Institutions, both private and public ones. The total amount of institutions is about 8,530, while institutions for health care are 4,500. Data are collected by a questionnaire sent by mail dealing with characteristics of people living in these Institutions (children, persons with disabilities, elderly), as well as of the services provided. In particular, the data include general information on the institution (identification data, type of management, organization of the institution), information on the personnel employed (number of persons with a specific profession by sex and type of contract), information on recipients (their number and distribution by gender, age-groups, nationality, type of needs) and economic information.

*Census Survey on intervention and social services provided by single and associated municipalities*.[D]: an annual survey that supplies information on users and charges of services supported by single and associated municipalities. The data are collected by a questionnaire divided in seven sections depending on the users’ typology: Family and Children, Disabled, Drug and alcohol addicted, Elderly, Immigrants and Nomads, Poverty and Adult discomfort, mixture of users.

*Resident population by age, gender and marital status* [E]: an annual survey providing data on resident population of single municipalities by gender, age and marital status.

*Hospital discharge register* [F]: an annually updated database that provides information on about 12 million of discharges from all public and private hospitals in Italy. It accesses information on demographic characteristics of patients (gender, age, place of birth, place of residence, nationality), on clinical patterns (main and secondary diagnosis and therapeutic procedures, length of stay), on organizational patterns (discharge ward, inpatient or day care).

*Home care for the elderly* [G]: data concerning home care for elderly were collected from FLS 21 form, one of the forms used in the annual statistical survey on Local Health Units (LHU) carried out by the Ministry of Health. The total amount of users of health home care is about 380,000, elderly population make up 85% of total users.

Data used in this study are retrieved from several national sources (administrative databases of Ministry of health and surveys of Istat) aiming to integrate different information on elderly.

This fragmentation of initial sources of data might pose some limitations but each of them can be properly dealt with.

Data can be "dirty". It is critical to remember that administrative data were gathered for other purposes than statistics. The fields we are most interested in may or may not be central to the primary record keeping. So missing data and lack of accuracy might be represented. Anyhow "cleaning" and data quality analysis, in addition to simple frequency distributions and cross tabulations of variables of interest, can identify the extent to which data in the field are complete and may reveal serious anomalies.

Furthermore, administrative data only help analysts partially understand the nature of the problem, and often cannot explain fully the underlying cause of the problem itself. Anyhow findings of the study carried out can be vital to policymakers in understanding how utilization patterns differ among population subgroups and pinpointing geographic areas within a community where problems are the most severe.

Another limitation is related to the integration of different data sources. The unit of analysis of the various datasets differs from individual to group level so that the integration could be difficult because of the lack of a common unit of analysis. Anyhow, a methodology performed by a multidisciplinary team (Epidemiologists, statisticians and health services research experts) helped in defining an adequate level of aggregation of data for the subsequent statistical analysis.
Finally regarding statistical sample surveys the main limitations are related to the sample size and to the design of sample survey. Both of these affect the sample errors and then the choice of unit of analysis, anyhow it is possible to take into account these sample errors to calculate the statistical accuracy of the estimation.
